# Natural relief for premenstrual syndrome (PMS): a double-blind clinical trial on the efficacy and safety of PMSoff

**DOI:** 10.1186/s40780-025-00495-6

**Published:** 2025-10-21

**Authors:** Fatemeh Saghafi, Parisa Zare, Nooshin Hatamizadeh, Maryam Malmir, Adeleh Sahebnasagh

**Affiliations:** 1 Department of Clinical Pharmacy, Faculty of Pharmacy and Pharmaceutical Sciences Research Center, Shahid Sadoughi University of Medical Sciences, Yazd, Iran; 2 Student Research Committee, Faculty of Pharmacy, Shahid Sadoughi University of Medical Sciences, Yazd, Iran; 3 Research and Clinical Center for Infertility, Yazd Reproductive Sciences Institute, Shahid Sadoughi University of Medical Sciences, Yazd, Iran; 4 Department of Chemistry, College of Science, University of Tehran, Tehran, Iran; 5 Clinical Research Center, Department of Internal Medicine , Faculty of Medicine, North Khorasan University of Medical Sciences, Bojnurd, North Khorasan Province Iran

**Keywords:** PMSoff natural supplement, Spirulina, Saffron, Premenstrual syndrome (PMS), Menstrual cycle, Double-blind clinical trial

## Abstract

**Background:**

Premenstrual syndrome (PMS) is a prevalent and often debilitating health conditions affecting women of reproductive age. The natural PMSoff supplement contains several active ingredients, including spirulina, whey protein, calcium citrate, vitamin B1, chamomile, turmeric, marigold, lavender, saffron, valerian, and aftimoon. This clinical trial aimed to assess the efficacy and safety of PMSoff, a natural supplement, in alleviating the symptoms of PMS.

**Methods:**

In this double-blind, randomized trial, women diagnosed with PMS were randomly assigned to receive either PMSoff or placebo. The primary objective of the study was to evaluate the impact of PMSoff on symptom severity, with secondary objectives focusing on safety and adherence. The primary outcome of this study was the severity of PMS symptoms, evaluated using the Daily Record of Severity of Problems (DRSP) questionnaire. The secondary outcome focused on the presence of premenstrual dysphoric disorder (PMDD), a more severe and debilitating form of PMS. Symptom severity was assessed at multiple time points: pre-intervention, one month post-intervention, and two months post-intervention.

**Results:**

Of the 255 randomized participants, 218 (85.4%) patients aged 14 to 30 years were enrolled and completed the clinical trial. No significant difference was observed between groups at baseline characteristics. Medication adherence in the first and second month of treatment was reported 72%. Following one month of treatment, the intervention group showed a significant reduction in DRSP scores compared to the control group (*P*-value = 0.043). This reduction became even more pronounced after two months of taking the supplement (*P*-value = 0.001). In patients with PMDD, a more severe form of PMS, a statistically significant difference emerged two months after the intervention (*P*-value = 0.04), indicating that the PMSoff was effective in alleviating PMDD-related symptoms, particularly during the second month of treatment.

**Conclusion:**

The results of this randomized placebo-controlled clinical trial were suggestive of the use of PMSoff in patients with PMS and PMDD to ameliorate its unpleasant symptoms, with sustained improvements observed over two months of treatment. Our findings suggest that PMSoff could be a viable alternative or adjunct to conventional pharmacological treatments for PMS and PMDD. Further studies are still demanded to explore the long-term effects, mechanisms of action, and broader applicability of this supplement.

**Trial registration:**

Trial Registry Date: 2025-03-02, Trial Registry number: IRCT20190810044500N30.

## Background

Premenstrual syndrome (PMS) is a prevalent and often debilitating condition that affects a substantial proportion of women during their reproductive years [1]. Among various countries, Iran reports the highest prevalence, with an estimated 98% of women experiencing PMS [2]. It is characterized by a range of physical, emotional, and behavioral symptoms that typically emerge during the luteal phase of the menstrual cycle and resolve with menstruation. The severity of these symptoms varies widely, ranging from mild discomfort to profound disruptions in daily functioning, significantly impairing the quality of life [3]. Women with PMS often experience diminished work productivity, increased absenteeism, and reduced participation in leisure activities and social engagements. Additionally, they tend to seek medical care more frequently due to the distressing nature of their symptoms [4].

Despite its widespread prevalence, the exact etiology of PMS remains uncertain, and ongoing research continues to explore its underlying mechanisms. One proposed hypothesis implicates dysregulation of the hypothalamic-pituitary-adrenal (HPA) axis, leading to adrenal hormone deficiencies that may contribute to PMS pathophysiology [5]. Furthermore, nutritional deficiencies and environmental influences have been suggested as potential contributing factors [6]. Emerging evidence also indicates that chronic inflammation and oxidative stress may play a role in PMS development [7]. Previous studies have demonstrated that women of reproductive age undergo cyclical changes in inflammatory status throughout the menstrual cycle, further supporting the involvement of inflammatory pathways in PMS [8].

Although PMS is commonly managed with pharmacological interventions such as nonsteroidal anti-inflammatory drugs (NSAIDs), hormonal therapies, and antidepressants, these treatments are often associated with side effects that may limit their long-term use and overall efficacy [9]. Consequently, there has been increasing interest in alternative medicine, particularly natural supplements, which may provide symptom relief without the adverse effects associated with conventional medications. The growing preference for natural remedies is driven by their perceived safety profile and the increasing demand for more patient-centered approaches to healthcare [10].

Numerous studies have investigated the use of herbal and natural therapies for managing PMS symptoms. One such natural supplement, PMSoff, is a combination of several active ingredients with potential therapeutic benefits. This formulation includes spirulina, whey protein, calcium citrate, vitamin B1, chamomile, turmeric, marigold, lavender, saffron, valerian, and aftimoon. Each of these components has been individually studied for their various health-promoting properties, including analgesic, anti-inflammatory, and mood-stabilizing effects. Spirulina, a nutrient-rich blue-green algae, has demonstrated potent anti-inflammatory and antioxidant properties, which may help alleviate PMS-related symptoms [11]. Whey protein, a rich source of essential amino acids, is believed to regulate hormonal fluctuations and may contribute to mood stabilization in women with PMS [12]. Calcium citrate is commonly used to alleviate bloating and mood swings, as calcium levels play a crucial role in maintaining hormonal balance throughout the menstrual cycle [13]. Vitamin B1 (thiamine) has been associated with improved mood and reduced anxiety, potentially benefiting women experiencing the emotional symptoms of PMS [14]. Chamomile and valerian, well-known herbal remedies, exhibit calming and sedative effects that may help alleviate insomnia and anxiety commonly reported by PMS sufferers [15, 16]. Additionally, turmeric, marigold, lavender, saffron, and aftimoon have demonstrated anti-inflammatory, analgesic, and antidepressant properties, further enhancing the therapeutic potential of this supplement [17, 18].

The combination of these ingredients in PMSoff appears to provide a comprehensive approach to managing PMS by addressing both physical and emotional symptoms. The safety of these components has been well-documented in previous studies, with no reported adverse effects when used at appropriate doses [19–26]. Given the natural origins of its active ingredients, PMSoff seems to be a promising alternative to conventional pharmacological treatments, particularly for women who experience inadequate symptom relief from traditional therapies. Therefore, the present study aimed to evaluate the efficacy and safety of PMSoff in alleviating premenstrual syndrome symptoms. To the best of our knowledge, this is the first randomized clinical trial investigating the use of PMSoff in patients with PMS.

## Materials and methods

### Study design, setting, and ethical considerations

This double-blind, randomized, parallel-group, placebo-controlled clinical trial was conducted over 13 months, from February 2024 to March 2025. The trial was carried out on patients aged 14 to 30 years old, recruited both from eight selected clinics, hospitals, and physician offices, as well as from the community through non-probability sampling with the study protocol approved by the Research Ethics Committee of Yazd University of Medical Sciences (Ethics Code: IR.SSU.MEDICINE.REC.1402.179). It should be stated that this study was registered in the Iranian Registry of Clinical Trials with registration number of IRCT20190810044500N30.

All participants were provided written informed consent before enrollment. For those under 18 years of age, parental or guardian consent was obtained. Participants were fully briefed on the study’s objectives, potential benefits, and risks and were assured of their right to withdraw at any time without repercussions. They were instructed to report any lack of symptom relief or the use of alternative pain management strategies. Although the adverse effects of these medicinal plants are generally mild and limited, in some individuals, side effects such as gastrointestinal disturbances, including nausea, vomiting, diarrhea or constipation, dry mouth, bloating, and heartburn, as well as appetite changes, headache, dizziness, drowsiness, and sleep disorders, may occur. Potential side effects of PMSoff were explained in detail, and participants were advised to contact a collaborating gynecologist if they experienced any adverse reactions.

### Participants

A total of 218 women, aged 14 to 30 years, were enrolled in the study based on a diagnosis of PMS confirmed by the Premenstrual Symptoms Screening Tool (PSST). Exclusion criteria included the presence of chronic illnesses, a history of pelvic inflammatory disease, ongoing medication use, and exposure to significant stressors, prior hysterectomy, psychological disorders, or lack of informed consent (including parental consent for participants under 18). The study protocol allowed participants to discontinue at any point without penalty, particularly in the presence of discomfort or perceived side effects.

### Randomization and blinding

Participants were recruited through two way: [1] patients referred to participating hospitals/clinics, and [2] community-based recruitment, for which non-probability approaches (snowball/convenience) were used due to limited access to the target population. Following recruitment, random allocation to intervention and control groups was applied and the participants were randomly assigned to either the intervention group, receiving the PMSoff supplement, or the control group, receiving a placebo. Randomization was conducted using a simple random allocation method by an independent individual not involved in the research team to ensure double blinding, so that neither participants nor researchers were aware of group assignments.

### Intervention

Participants in the intervention group received PMSoff, a natural supplement formulated with herbal extracts, vitamins, and minerals. Each capsule contained the following ingredients: lavender extract (50 mg), lesser dodder extract (90 mg), valerian extract (40 mg), spirulina extract (40 mg), saffron extract (13 mg), curcumin (10 mg), chamomile extract (25 mg), rose extract (100 mg), calcium citrate (50 mg), and vitamin B1 (56 mg). Participants were instructed to take the supplement twice daily for the duration of the study. Participants were instructed to begin supplementation seven days before their expected menstruation and continue for three days after menstruation onset, maintaining this regimen for three consecutive menstrual cycles. The control group received an identical placebo capsule, devoid of active ingredients and distinguishable only by a serial number. Both oral and written instructions, including an informational brochure, were provided to ensure proper adherence to the prescribed regimen.

### Study procedures

Upon enrollment, participants completed the DRSP questionnaire for two consecutive menstrual cycles to establish baseline PMS symptom severity. Demographic and clinical data were also collected at baseline. Throughout the study, participants maintained a symptom diary and completed the DRSP questionnaire for two menstrual cycles before and three cycles after the intervention.

To enhance compliance and monitor adherence, bi-weekly phone calls were conducted. Participants were instructed to refrain from using other treatments or supplements during the study period. The use of additional pain medications, including acetaminophen, Mefenamic acid, or other NSAIDs, was recorded and incorporated into the final analysis.

#### Study timeline

The study was conducted in two phases:

- Phase 1: The first assessment occurred one month after the start of the intervention, where PMS symptoms were measured using the DRSP questionnaire.
- Phase 2: The second assessment took place two months after the start of the intervention, where the same symptom measurements were conducted.

Participants were instructed to maintain their usual dietary and physical activity habits throughout the study to ensure that any observed effects could be attributed to the intervention rather than lifestyle changes.

### Measures

The primary outcome of the study was the severity of PMS symptoms, assessed using the DRSP questionnaire, which evaluates symptoms across five domains: anxiety, depression, emotional symptoms, physical symptoms, and water retention. The secondary outcome was the presence of PMDD, a more severe form of PMS. Treatment adherence was assessed using the Morisky Medication Adherence Scale.

### Tools for data collection

Data were gathered using the following validated questionnaires:

- Demographic Questionnaire: A researcher-designed tool used to collect participant demographic information, including age, body mass index (BMI), educational level, and marital status, number of children, menstrual cycle characteristics, and family history of PMS.
- Premenstrual Symptoms Screening Tool (PSST): A diagnostic screening tool developed in 2003 to assess the presence and severity of PMS. It consists of 19 items covering mood, behavioral, and physical symptoms, with responses categorized on a 4-point scale (none, mild, moderate, severe). The PSST has been validated in multiple studies [27, 28].
- Daily Record of Severity of Problems (DRSP): A standardized instrument for tracking PMS symptoms over two menstrual cycles. It categorizes symptoms into five domains (anxiety, depression, emotional symptoms, physical symptoms, and water retention) and is used to assess symptom changes over time [29, 30].

### Sample size

The required sample size was determined to detect 25% improvement in PMS symptom severity in the intervention group compared with the control group [31]. Using a two-sided significance level (α) of 0.05 and a statistical power (1 − β) of 0.95, the sample size was estimated based on the prevalence of PMS symptoms in the general population and the formula for comparing two proportions:

N= (pi​−pc) 2(Z1 − α/2​+Z1 − β) 2[pc​(1 − pc) + pi​(1 − pi)]​

This calculation yielded a sample size of approximately 105 participants per group. To account for an anticipated dropout rate of 10%, the final sample size was increased to 115 participants per group, ensuring sufficient power to detect statistically significant differences between the intervention and control groups.

### Statistical analysis

Descriptive statistics were used to summarize participant characteristics and PMS symptom severity. Continuous variables were expressed as means and standard deviations, while categorical variables were presented as frequencies and percentages. The Chi-square test was used to assess associations between categorical variables.

For group comparisons, independent sample t-tests and paired sample t-tests were conducted to evaluate differences in PMS symptom severity before and after the intervention between the PMSoff and placebo groups. One-way repeated measures ANOVA was employed to analyze changes in symptom severity across multiple time points (pre-intervention, one month post-intervention, and two months post-intervention). Between-group comparisons were performed using analysis of covariance (ANCOVA) to adjust for baseline values. All the statistical analysis was conducted by Statistical package for social science (SPSS) software version 23 and *P*-value < 0.05 was considered as statistically significant.

## Results

### Study participants

Initially, 340 patients were evaluated for eligibility. However, 85 individuals were excluded prior to randomization for the following reasons: 32 did not meet the diagnostic criteria for PMS, 28 declined participation, 14 patients had irregular menstrual cycles, 5 patients were breastfeeding and 6 cases were excluded due to pregnancy. The remaining 255 participants were randomly assigned to either the intervention (PMSoff) or the control groups.

During the study period, several participants withdrew or were excluded for various reasons: 22 participants withdrew due to a lack of willingness to continue (16 from the control group and 6 from the intervention group), 6 participants were lost to follow-up due to non-responsiveness and failure to attend scheduled monthly visits (3 from each group), 4 participants were excluded following pregnancy and subsequent miscarriage (1 from the control group and 3 from the intervention group), 4 participants in the intervention group withdrew due to concerns about potential drug interactions, and 1 participant in the control group was excluded after being diagnosed with cervical cancer.

**Fig. 1:**
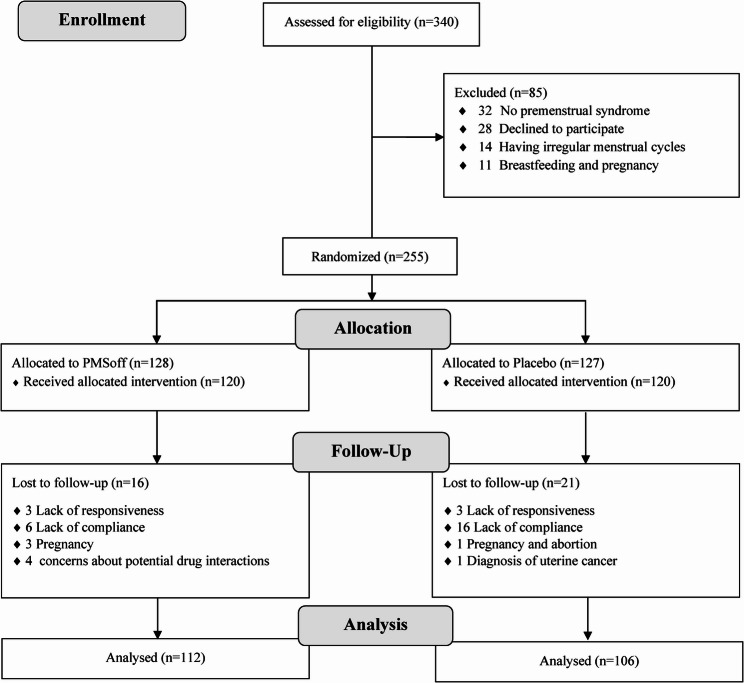
Flow diagram of the study

Ultimately, 218 participants with a mean [SD] age of 27.3 [3.9] years completed the study and were included in the final analysis (106 in the control group and 112 in the intervention group). A CONSORT flow diagram illustrating participant allocation and retention is provided below (Fig. 1).

### Demographics

Table 1 provides an overview of the baseline characteristics of the 218 participants enrolled in the study, divided into two groups: the intervention group (*n* = 112), which received PMSoff and the control group (*n* = 106), which received the placebo. Of the total study population, 67 (30.9%) participants were single and 151 (69.1%) were married. Regarding the educational status of the study population, 14 (6.4%) patients had primary/secondary education, 69 (31.7%) cases had high school education, 102 (46.8%) patients had diploma/bachelor’s degree and 33 (15.1%) of participants had master’s degree and above. Furthermore, 159 (72.9%) patients had a positive family history for PMS and 34 (15.6%) had history of medication use and Dysmenorrhea was presence in 208 (95.4%) of patients. Both groups were comparable across demographic variables, menstrual cycle characteristics, and premenstrual symptom severity prior to the intervention.

**Table 1:** Baseline characteristics of participants in the intervention and control groups

Variable	Intervention Group (*n* = 112)	Control Group (*n* = 106)	*P*-Value	Statistical Test
Age (years, mean ± SD)	27.52 ± 3.5	26.56 ± 3.8	0.52	Independent Sample T-test
Number of Children (mean ± SD)	1.84 ± 0.61	1.69 ± 0.58	0.175	Fisher’s Exact Test
Height (cm, mean ± SD)	163.25 ± 6.3	162.78 ± 5.9	0.650	Independent Sample T-test
Weight (kg, mean ± SD)	67.38 ± 10.5	65.06 ± 8.9	0.083	Independent Sample T-test
BMI (kg/m², mean ± SD)	25.22 ± 3.6	24.43 ± 3.09	0.085	Independent Sample T-test
Menstrual Cycle Characteristics (days, mean ± SD)				
- Duration of Menstruation	6.82 ± 1	6.84 ± 1	0.907	Independent Sample T-test
- Bleeding Days	3.63 ± 1.2	3.69 ± 1.5	0.772	Independent Sample T-test
- Cycle Length	28.07 ± 2.44	27.74 ± 3.0	0.151	Independent Sample T-test
Educational status, N (%)				
-Primary/secondary education	5(4.5)	9(8.5)	0.655	Chi-square test
-High school education/Diploma	34(30.4)	35(33)		
- bachelor’s degree	55(49.1)	47(44.3)		
-Master’s degree and above	18(16)	15(14.2)		
Marital status, N (%)				
-Single	31(27.7)	36(34)	0.315	Chi-square test
-Married	81(72.3)	70(66)		
Family history, N (%)				
-PMS, yes	85(75.9)	74(69.8)	0.312	Chi-square test
-Dysmenorrhea, yes	109(97.3)	99(93.4)	0.166	Chi-square test
history of medication use	20(17.9)	14(13.2)	0.344	
Pain and Symptom Severity, (mean ± SD)				
- Severity of Menstrual Pain^1^	3.94 ± 1.13	3.64 ± 1.16	0.056	Independent Sample T-test
Severity of Bleeding	2.52 ± 0.75	2.45 ± 0.68	0.502	Independent Sample T-test
PMS Symptom Severity	29.6 ± 8.0	27.8 ± 7.9	0.104	Independent Sample T-test

^1^Pain was assessed using the Visual Analog Scale (VAS), where participants indicated their pain severity from 0 to 10

The mean age of participants in the intervention group was 27.52 ± 3.5 years, while the control group had a mean age of 26.56 ± 3.8 years (*P*-value = 0.52). The majority of participants were married (69.1%), with no significant difference in marital status between the two groups (*P*-value = 0.315). The other baseline characteristics, including BMI, menstrual cycle length, duration of bleeding, and family history of PMS, were similar between the two groups (*P*-value > 0.05 for all comparisons), suggesting that the randomization process was successful. In terms of menstrual pain and symptom severity, 95.4% of participants reported dysmenorrhea, with no significant difference between groups (*P*-value = 0.165). The severity of PMS symptoms before the intervention was also comparable, with the intervention group reporting a mean DRSP score of 29.6 ± 8.0, compared to 27.8 ± 7.9 in the control group (*P*-value = 0.104).

The absence of statistically significant differences at baseline characteristics indicates that any observed changes following the intervention could be attributed to the effects of PMSoff rather than confounding factors.

In terms of additional analgesic use, 42.5% of participants in the control group and 43.8% in the intervention group did not use any analgesics during the study. A single analgesic was used by 40.6% of participants in the control group and 37.5% in the intervention group, while two analgesics were consumed by 16.0% and 16.1% of participants in the control and intervention groups, respectively. A small proportion of participants (0.9% in the control group and 2.7% in the intervention group) reported use of three analgesics throughout the study period. As presented in Table 2, the comparison of these distributions using the chi-square test revealed no statistically significant difference between the two groups (*P*-value = 0.79).

**Table 2:** Additional use of analgesics during the study

The number of consumed analgesics during the study period	Intervention Group (*n* = 112)	Control Group (*n* = 106)	*P*-Value
No consumed analgesics n (%)	49 (43.8%)	45 (42.5%)	0.79
One consumed analgesic n (%)	42 (37.5%)	43 (40.6%)	
Two consumed analgesics n (%)	18 (16.1%)	17 (16.0%)	
Three consumed analgesics n (%)	3 (2.7%)	1 (0.9%)	

### Distribution of respondents’ answers regarding medication adherence (Morisky medication adherence Questionnaire)

#### First month

In the control group, 67% of participants adhered to their prescribed regimen, while 33% missed doses. In the intervention group, 76.8% adhered, and 23.2% missed doses. The difference between groups was not statistically significant (*P* = 0.107). A very small proportion of participants (< 1.5%) discontinued medication without medical consultation due to perceived worsening of symptoms or unspecified reasons.

#### Second month

Adherence remained similar, with 68.9% of the control group and 71.4% of the intervention group following their regimen as prescribed. Missed doses occurred in 31.1% and 28.6% of participants, respectively. No statistically significant difference was observed between groups (*P* = 0.68). Again, a small fraction of participants (< 1%) discontinued medication without medical consultation.

### Study outcomes

#### Effect of PMSoff on PMS symptom severity

Table 3 represents the results of statistical analyses assessing the effect of the PMSoff supplement on PMS symptom severity over time. Symptom severity was evaluated using the DRSP questionnaire at three time points: One month before the intervention, one month after the intervention, and two months after the intervention

**Table 3:** Effect of PMSoff on PMS symptom severity using DRSP score

Time Point			Control	Intervention	*P-Value*	Statistical Test
			Mean ± SD			
DRSP Score	Overall	1 Month Before Intervention	37.86 ± 23.07	43.28 ± 18.99	0.06	Independent Sample T-test
		1 Month After Intervention	30.79 ± 15.8	26.2 ± 17.3	0.043	Independent Sample T-test
		2 Months After Intervention	28.61 ± 13.9	21.02 ± 18.45	0.001	Independent Sample T-test
	PMDD Patients	1 Month Before Intervention	44.94 ± 22.4	48.26 ± 19.01	0.356	Independent Sample T-test
		1 Month After Intervention	41.25 ± 17.47	44.83 ± 29.84	0.410	Independent Sample T-test
		2 Months After Intervention	36.27 ± 17.27	30.13 ± 17.48	0.044	Independent Sample T-test

Overall *N* = 218 PMDD *N* = 134 (intervention = 73(65.2), placebo = 61(57.5))* P*-Value = 0.247

### Comparison between intervention and control groups

Before the intervention, there were no significant differences in DRSP scores between the intervention and control groups (*P*-value = 0.06). However, following one month of treatment, the intervention group showed a significant reduction in DRSP scores compared to the control group (*P*-value = 0.043). This reduction became even more pronounced after two months, with the intervention group experiencing a much greater decrease in symptom severity (*P*-value = 0.001).

### Within-Group comparisons over time

In the control group, symptoms decreased over time, and the reduction was clinically significant. The mean DRSP score dropped from 37.86 ± 23.07 before the intervention to 30.79 ± 15.82 one month after and 28.61 ± 13.9 two months after (*P*-value = 0.001). Similarly, the intervention group experienced a significant and sustained reduction in symptom severity. The mean DRSP score dropped from 43.28 ± 18.99 before the intervention to 26.2 ± 17.32 after one month and 21.02 ± 18.4 after two months (*P*-value = 0.001). The results of the analysis of covariance (ANCOVA) indicated that the dependent variable (PMS symptom severity) was comparable between the intervention and control groups at baseline (*P*-value = 0.508). It should be noted that the reduction in the intervention group was significantly greater than in the control group (*P*-value = 0.001) (Tables 3 and 4).

**Table 4:** Within-Group comparisons of DSRP score over time

Groups	Time Points		Mean difference (SD)	*P*-Value
Control	1 Month Before Intervention	1 Month After Intervention	7.07 (1.8)	0.001
		2 Months After Intervention	9.25 (1.84)	0.001
	1 Month After Intervention	2 Months After Intervention	2.18 (1.27)	0.089
Intervention	1 Month Before Intervention	1 Month After Intervention	17.08 (1.75)	0.001
		2 Months After Intervention	22.25 (1.79)	0.001
	1 Month After Intervention	2 Months After Intervention	5.17 (1.24)	0.001

One-Way Repeated Measures ANOVA was used

Although symptom severity decreases over time in both the control and intervention groups, the reduction in the intervention group is significantly more pronounced. The mean severity of PMS symptoms in the intervention group decreases to a level below that of the control group. The gap between the control and intervention groups, as shown in the graphs two months after the intervention, highlights the substantial difference in symptom reduction.

Although symptoms decreased in both the control and intervention groups, the intervention group experienced a significantly more pronounced reduction in symptom severity. By the second month, the mean severity of PMS symptoms in the intervention group was significantly lower than that of the control group.

#### Effect on PMDD patients

As presented in Table 3, the mean severity of PMS symptoms in patients diagnosed with premenstrual dysphoric disorder (PMDD) within the control group was 44.94 ± 22.4 one month before the intervention, 41.25 ± 17.5 one month after the intervention, and 36.27 ± 17.3 two months post-intervention. In contrast, the intervention group exhibited a mean symptom severity of 48.26 ± 19.01 before the intervention, which decreased to 44.83 ± 29.8 one month post-intervention and further declined to 30.13 ± 17.5 at the two-month follow-up.

Statistical analysis using the Independent Sample t-test revealed no significant difference in PMS symptom severity between the control and intervention groups before the intervention (*P*-value = 0.36) or at the one-month follow-up (*P*-value = 0.41). However, a statistically significant difference emerged two months after the intervention (*P*-value = 0.04).

Among the final 218 participants who completed the study, no serious adverse events were reported. Minor side effects included gastrointestinal disturbances, such as nausea (*n* = 2), bloating (*n* = 1), and heartburn (*n* = 3), as well as appetite changes (*n* = 2). All of these adverse events were transient and did not necessitate withdrawal from the study.

## Discussion

In this placebo-controlled double-blinded clinical trial, the therapeutic effect of PMSoff, a natural supplement, was investigated in reducing the severity of PMS and PMDD symptoms. The results of the present study demonstrated that PMSoff is effective in reducing overall PMS severity, and was also effective in patients diagnosed with PMDD. Our study therefore offers evidence supporting the therapeutic potential of PMSoff in the management of PMS.

The results of the present trial showed a significant reduction in DRSP scores, particularly in the intervention group both in the first and second months of treatment. Furthermore, the ANCOVA results confirmed the superiority of PMSoff compared to placebo in relieving PMS symptoms. By adjusting for baseline DRSP scores, the ANCOVA results demonstrated that patients in the intervention group had significantly greater improvement in symptom severity than those in the placebo group. This adjustment of baseline DRSP scores is crucial, since it allows for baseline balance and between-group comparisons. This represents that the observed within-group differences are not simply attributed to initial changes in symptom severity, but rather reflect an actual therapeutic effect of the PMSoff supplement and a sustained improvement in symptom burden over the two-month follow-up, supporting the robustness of the intervention’s effectiveness. A comprehensive systematic review and meta-analysis examined the efficacy and safety of herbal medicine and nutritional supplements in alleviating premenstrual somatic and psycho-behavioral symptoms. Their results showed that these interventions can significantly reduce PMS symptoms and are relatively safe [32]. Additionally, a recent systematic review focused on nutritional practices for managing menstrual cycle-related symptoms. The findings suggested that herbal and nutritional supplements such as vitamin D, calcium, zinc, and curcumin may help reduce symptoms associated with PMS [33]. Similarly, Ebrahimi et al. found that a supplement of magnesium and vitamin B6 significantly reduced PMS symptoms, after four months of treatment [34].

As stated, PMSoff contains a combination of active ingredients to which symptom improvement was attributed. Unlike other studies that used single-component interventions (e.g., magnesium or vitamin B6), PMSoff may exert a synergistic effect through its multi-ingredient formulation, potentially targeting different pathways involved in PMS symptomatology, such as inflammatory cytokines, hormonal fluctuations, serotonin modulation, and neurochemical regulation, targeting both the psychological and physical components of PMDD. However, some studies have reported modest effects of natural supplements on PMS symptom severity. For instance, in a clinical trial by Mirghafourvand et al., the efficacy of lemon balm alone or in combination with Nepeta menthoides was investigated on PMS and menstrual bleeding. Their results suggested that this herbal supplement could reduce the severity of PMS symptoms, with no significant effect on menstrual bleeding in women in reproductive age [35, 36]. Similar to our study, they screened patients for the presence and severity of PMS with the DRSP questionnaire, and the intervention and follow-up continued for two consecutive menstrual cycles and two luteal phases.

The present RCT also explored the effects of PMSoff on individuals with PMDD, a severe form of PMS characterized by significant mood disturbances and functional impairment [37]. In our study, participants with PMDD in the intervention group experienced a substantial reduction in symptom severity, particularly at the two-month follow-up. These findings are consistent with those of the study by Su Hee Jang et al. In this systematic review on the efficacy of acupuncture and herbal medicine treatments for premenstrual syndrome and PMDD, a 50% or greater reduction in symptoms was reported compared to baseline [38]. It appears that complementary and alternative medicine may offer a substantial relief for individuals experiencing severe symptoms of PMS, including those characterized as PMDD.

Medication adherence is a critical factor in assessing treatment efficacy. In our study, 72% of participants in both groups reported adherence to their treatment regimens. The lower rate of discontinuation in our study may reflect the generally good tolerability of PMSoff. Furthermore, additional analgesic use could potentially influence pain outcomes. However, the proportion of participants who consumed analgesics during the study did not differ significantly between the intervention and control groups. This indicates that the use of additional analgesics including acetaminophen, mefenamic acid, and other NSAIDs was balanced between groups, and therefore unlikely to have confounded the observed study outcomes.

The findings of this study underscore the potential clinical utility of PMSoff as an alternative to traditional pharmacologic treatments for PMS and PMDD. While pharmacologic options, such as SSRIs and oral contraceptives, are commonly used to manage severe PMS and PMDD, they often come with undesirable side effects, including weight gain, mood swings, and sexual dysfunction [39, 40]. In contrast, natural supplements like PMSoff may provide a safer and more tolerable option for patients seeking symptom relief without the associated risks of pharmacological therapies.

The favorable safety of PMSoff makes it a promising alternative for women with PMS and PMDD who are dissatisfied with conventional treatment options. While the results of this clinical trial were encouraging, there were limitations that should be addressed in future research. One limitation was the lack of long-term follow-up information beyond two months. The beneficial effects of natural supplements for PMS may continue to improve over time, suggesting that longer treatment periods may yield even greater symptom relief. Future trials should assess the sustained benefits of PMSoff over a more extended period, particularly in relation to long-term adherence and any potential rebound effects after discontinuation. Furthermore, the generalizability of these findings could be improved by including a more diverse population with varying ethnic backgrounds and geographic locations. Moreover, the use of snowball sampling for initial sampling, a non-probabilistic recruitment method, may limit the representativeness of the sample and thus the generalizability of the results. However, once eligible participants were enrolled, random allocation to intervention and control groups was applied, to ensure baseline comparability. Future studies should employ probabilistic sampling methods to enhance sample representativeness. Additionally, future studies should explore the mechanisms by which PMSoff exerts its therapeutic effects, including its effects on hormonal regulation, inflammatory pathways, and neurotransmitter modulation to provide a more comprehensive assessment of its biological basis. By investigating these pathways, it is better understood how PMSoff works at the molecular level and refine its formulation for even greater therapeutic effect. Finally, a detailed item-level analysis of changes in individual items of the DRSP questionnaire to differentiate the effects of the intervention on psychological and physical domains was not performed in this study, which should be addressed in future research to provide a more comprehensive understanding of PMSoff’s impact.

## Conclusions

The results of this randomized placebo-controlled clinical trial were suggestive of the use of PMSoff in patients with PMS in ameliorating the unpleasant symptoms of PMS and PMDD, with sustained improvements observed over two months of treatment. This intervention showed significant benefits, particularly for psychological symptoms, and was well-tolerated by participants with high rates of adherence. The findings suggest that PMSoff could be a viable alternative or adjunct to conventional pharmacological treatments for PMS and PMDD, and could serve as a promising option for individuals seeking a natural therapeutic approach. However, further studies are still demanded to explore the long-term effects, mechanisms of action, and broader applicability of this supplement.

## Data Availability

All data generated or analyzed during this study are included in this published article.

## Abbreviations

PMS: Premenstrual yndrome
HPA: Hypothalamic-pituitary-adrenal
NSAIDs: Nonsteroidal anti-inflammatory drugs
PSST: Premenstrual symptoms screening tool
DRSP: Daily Record of Severity of Problems
PMDD: Premenstrual dysphoric disorder
SPSS: Statistical package for social science
VAS: Visual Analog Scale
